# Cancer in Japan: Prevalence, prevention and the role of heterocyclic amines in human carcinogenesis

**DOI:** 10.1186/s41021-016-0043-y

**Published:** 2016-07-01

**Authors:** Minako Nagao, Shoichiro Tsugane

**Affiliations:** National Cancer Center Research Institute, Tokyo, Japan; Center for Public Health Sciences, National Cancer Center, Tokyo, Japan

**Keywords:** Cancer in Japan, Prevention, Infection, Tobacco smoking, Alcohol, Heterocyclic amines

## Abstract

In this article, three topics are being studied in order to understand the present state of cancer in Japan. First, the statistics on cancer mortality indicates that the mortality from cancer in young individuals has been decreasing during the last 50 years, although the total mortality from cancer has been steadily and steeply increasing. Second, epidemiological analyses of cancer causes in Japan indicated that 50 % of cancer cases are preventable, and that prevention of infection and refraining from tobacco smoking will reduce cancer mortality by about 40 %. Third, mutagenic/carcinogenic heterocyclic amines present in cooked meat/fish have been suggested to be carcinogenic in humans in many epidemiological studies carried out in Japan and other countries.

## Background

Cancer is the leading cause of death in Japan for more than 30 years, and mortality rate is further increasing year by year. On the other hand tremendous efforts have been made in clarification of etiology, prevention and treatment of cancers. It is important to clearly understand the reason for this apparently contradictory phenomenon. In this article we summarized chronological changes of mortality rates of cancer in Japan, taking the age factor into consideration. Data are based on the report of Cancer Statics in Japan 2014 [1].

Many cancer etiology studies have been done on US or European people, however, study on Japanese people was reported, only quite recently, by one of the authors’ (Tsugane’s) group. The study results suggested that 50 % of cancers are preventable.

Importance of dietary habit in cancer development has been pointed out by epidemiological studies [2], and one of the authors’ (Nagao’s) group detected presence of mutagenic/carcinogenic heterocyclic amines (HCAs) in heated fish and meats [3]. Recently, many epidemiological studies have been performed in the United States, Europe and Japan to clarify the roles of HCAs in human carcinogenesis, and involvement of HCAs in human carcinogenesis is suggested.

Information provided in this article will be helpful for strategy for prevention of cancer.

## Chronological changes in cancer mortality in Japan

**Fig. 1:**
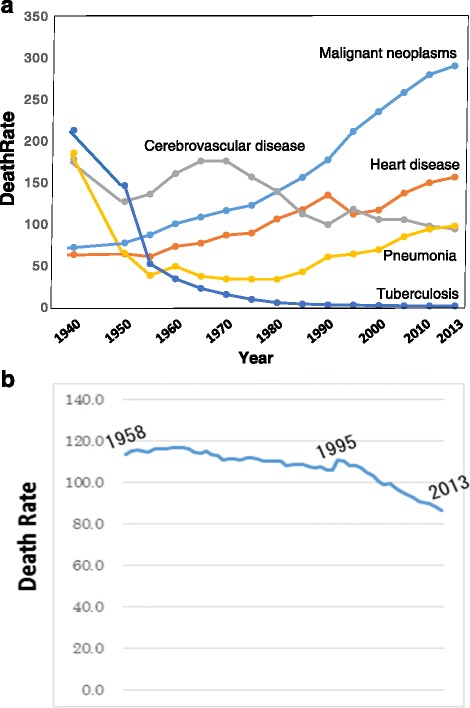
**a** Trend in overall mortality rate for leading causes of death in Japan [1]. **b** Trend in age-adjusted mortality rate of cancer in Japan [1]

**Fig. 2:**
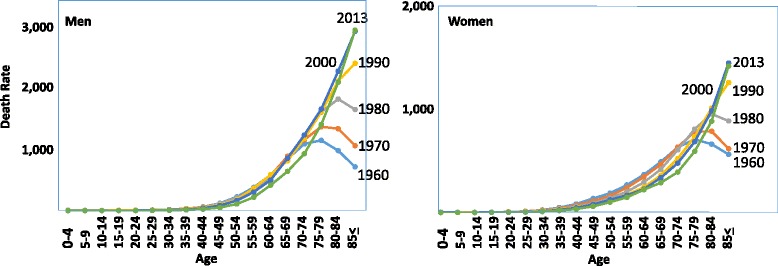
Trend in age-specific mortality rate of cancer in Japan. Men and women [1]

The mortality from cancer in Japan has been steadily and steeply increasing since 1947, and cancer has become the foremost cause of death since 1977, followed by heart disease, cerebrovascular disease and pneumonia (Fig. 1a) [1]. However, the age-adjusted mortality (adjusted to the age-structure in 1985) has been decreasing dramatically since 1995 (Fig. 1b), and in 2013, a 22 % reduction compared to the rate in 1995 was observed [1]. Chronological changes in age-specific mortality are shown in Fig. 2 [1]. The data indicate that the cancer mortality in both men and women has been decreasing among younger people, while it has been increasing in older people during the last 50 years. These data indicate that the increase in cancer mortality observed in the total population (Fig. 1a) is due to an increase in the lifespan, since the lifespan is increasing in Japan, and the average lifespans are 81 years in men and 86 years in women in 2013. Based on the mechanisms of cancer development, it would be natural to suffer from cancer at an old age, because humans are exposed to various weak environmental carcinogens during their whole lifetime and experience spontaneous DNA replication errors. The present situation in Japan is steadily on the way to our goal of preventing death from cancer in young people.

The data from a national survey indicate that various strategies for cancer prevention might contribute to a reduction in cancer mortality in young individuals. Of course, developments in cancer diagnosis and treatment also play important roles in prevention of cancer deaths in young individuals. However, there are still many preventable cancers. In the next section, we discuss how it will be possible to prevent cancer in the Japanese population.

## Cancer causes in Japan

One of the authors’ (Tsugane’s) group studied the causes of cancer based on the data on 647,000 cancer patients and 326,000 cancer deaths in 2005 [2]. About 15 years are required for development of cancer after exposure to a cause, so it is suspected that the exposure to various causes took place around 1990. The investigators analyzed the effects of risk factors that were classified by the International Agency for Research on Cancer (IARC) as Group 1 carcinogens in humans [4]. They also analyzed the risk and protective factors that were judged as ‘convincing’ or ‘probable’ in the second expert report on “Food, nutrition, physical activity, and the prevention of cancer” by the World Cancer Research Fund and American Institute for Cancer Research in 2007 [5]. The conditions evaluated by the IARC and reported in the Cancer Prevention Handbook Series [6] as causally associated with a reduced risk were also analyzed.

Involvement of tobacco smoking was demonstrated in various cancers. The population attributable fraction (PAF) of tobacco smoking to cancer was 24 %, infection was 22 %, alcohol drinking was 6 %, and over-intake of salt was 1.4 %. Overweight, less-intake of vegetables and fruits, less amount of exercise, use of exogenous hormones were less than 1 %, respectively. Effects of UV and radiation exposure, air pollution, occupational exposure and genetic effects were not studied. Occupation and genetic effects were estimated to be about 5 %, respectively. Contributions of the former three (UV, radiation and air pollution) may not be large. In total, we were able to deduce the causes of about 50 % of cancer deaths. The causes of the other 50 % of cancer death were not sufficiently clarified. Tsugane’s group also studied PAF for cancer incidence and found that it is almost the same as that for cancer death [2].

Table 1 summarizes organ specific mortality and epidemiologically detected causes of cancer.

**Table 1:** Organ specific death rate per 10^5^ and major risk factor in 2005 [1, 2]

Organ	Death rate	Risk factor
Lung	49.2	Smoking
Stomach	39.9	H. pylori, Smoking, Salt,
Colorectum	32.4	Alcohol drinking, Smoking, Overweight, Physical activity,
Liver	27.2	HCV, HBC, Alcohol drinking, Smoking,
Pancreas	18.2	Smoking
Breast	16.6	Alcohol, Overweight
Prostate	15.0	
Bladder, bile duct	13.1	
Kidney,Urater, Urinary bladder	9.6	Smoking, Overweight,
Uterus	8.3	HPV
Esophagus	8.9	Smoking, Alcohol drinking
Ovary	6.9	
Oral cavity, Pharynx	4.5	Smoking, Alcohol drinking

### Tobacco smoking

In 1965, 84 % of Japanese men smoked, however, the number of smokers decreased, and it was down to 30 % in 2014 [7]. About 10–15 % of Japanese women smoked during these periods. Tobacco smoking plays a major role as a cause of cancer in the lung, larynx, oral cavity, pharynx, renal pelvis and bladder. It is also involved with colorectal, liver, pancreas and kidney carcinogenesis (Table 1). Relative risks higher than 2 of tobacco smoking were detected in the following organs; larynx (4.5 for men, no data is available for women), lung (3.85 for men and 3.55 for women), esophagus (2.96 for men and 2.40 for women), oral pharynx (2.37 for men and 1.76 for women) and renal pelvis, ureter and bladder (4.30 for men and 1.30 for women). Liver (1.75 for men and 1.59 for women), pancreas (1.43 for men and 1.85 for women) and stomach (1.42 for men and 1.29 for women) cancer risks are also affected by tobacco smoking.

### Infection

**Fig. 3:**
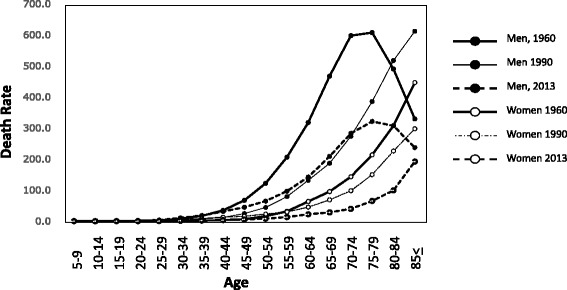
Trend in age-specific mortality rate of stomach cancer [1]

As for infection, *Helicobacter pylori* is involved in non-cardia gastric cancer in Japan. A total of 81.5 % of men and 69.9 % of women who died from non-cardia stomach cancer and 75.7 % of stomach MALT (mucosa-associated lymphoid tissue) lymphoma deaths in men and women were assigned to be due to *H. pylori* infection. The relative risk of *H. pylori* infection in gastric cancer development is 6.8 for men and 4.6 for women [2]. In 1950, about 80 % of adults were infected with *H. pylori*, but in 2010, less than 20 % of individuals younger than 40 were infected. Since no special precautions were made, the decrease in infection rate in young people may be due to the improvement of general hygienic conditions in Japan [8, 9]. Further, eradication of *H. pylori* is covered by the national medical insurance. It is also evident that the mortality from stomach cancer is decreasing in both men and women since 1970 [1] (Fig. 3).

As for liver cancer in Japan, the major causes are hepatitis viruses. A total of 12.5 % of liver cancer deaths is due to hepatitis B virus (HBV), 72 % to hepatitis C virus (HCV) and 1.8 % to HBV plus HCV. The relative risks of HBV, HCV and HBV plus HCV infection were 102, 126 and 572, respectively [2]. HBV infection is mainly transmitted from mother to child. Vaccination against HBV prevents infection. Infection by HCV is mainly due to blood transfusion and inappropriate use of medical equipment. Almost no infection occurs from mother to child. Therefore, infection by HCV has decreased dramatically, and only 0.2 % of 20–29-year-olds are infected in Japan. New antiviral agents against HCV have been developed, and they are now available to patients in addition to interferon treatment. Based on this evidence, it can be said that most hepatic cancer development will be prevented in the near future.

All of the uterus cervical cancers are due to infection by human papilloma virus (HPV). Therefore, the relative risk of HPV is infinite. Infection is due to sexual activity. The mortality from cervical cancer decreased in 1995 in comparison to that in 1992. However, it was increasing after 2000, and in 2013 the mortality in young individuals, 20 to 40 years old, had increased. In Japan, vaccines against HPV 16/18 and HPV 6/11/16/18 are used. Vaccination is targeted to high school students, and the rate of vaccination was 67.2 % in 2012. However, in 2013, side effects were reported, and the Ministry of Health, Labour and Welfare in Japan has temporarily suspended encouragement to be vaccinated.

It was found in Japanese studies that human adult T cell leukemia virus type 1 (HTLV-1) induced adult T cell leukemia (ATL) [10]. About 108 million people infected with this virus were reported by the Ministry of Health, Labour and Welfare in Japan in 2009, mainly from the south-west part of Japan. The ATL-inducing rate by HTLV-1 is not high, and about 1,100 die every year. ATL is only caused by this virus, and the relative risk is infinite. Twenty percent of virus carriers are considered to be infected through breast milk feeding.

### Alcohol drinking

Alcohol drinking is involved with development of cancers in colon, liver, esophagus oropharynx and breast. The relative risks and PAF in these organs from drinking alcohol are summarized in Table 2. Many Japanese have a genetic defect in metabolisms of alcohol. Aldehyde dehydrogenase 2 (ALDH2) has a polymorphism at codon 504 where Glu or Lys is encoded. Glu but not Lys has dehydrogenase activity. Fifty percent of Japanese people have the Lys allele. Heterozygous people easily get flushed by drinking alcohol, and there is a strongly active form in alcohol dehydrogenase (ADH). There are at least 7 forms in ADH, ADH1A, ADH1B, ADH1C, TADH4, ADH5, ADH6 and ADH7 [11]. If ADH1B is the active form, the subject would get flushed even with full activity of Glu/Glu ALDH. The mortality due to drinking alcohol is minimized at 46 g/day for men and 23 g/day for women [2].

**Table 2:** Rerative risk and PAF of alcohol drinking [2]

Organ	RR		PAF (%)	
	men	women	men	women
Oral cavity, pharynx	2.02	2.02	43.9	21.5
Esophagus	2.52	2.52	53.8	28.9
Colorectum	1.64	1.08	32.9	2.1
Liver	1.19	1.90	11.6	12.3
Breast		1.22		5.6

### Overweight and salt intake

Overweight also contributes to induction of cancer. The Japanese body mass index (BMI) is not high and only about 25 % of men and women have more than 25 of BMI. A total of 15 % of corpus uterus cancer in women was judged as being due to obesity with a relative risk of 1.73. Also, 13.2 % and 12.0 % of kidney cancer, and 5.2 and 4 % of colon cancers in men and women, respectively, are also due to obesity.

The salt intake of the Japanese is evidently decreasing, it was 10.9 g/day in men and 9.2 g/day in women in 2014 [6], but it is still high from the point of view of prevention of stomach cancer. By assuming that 6 g/day of salt has no inducing effect on stomach cancer, 8.91 % of stomach cancer was attributed to excess intake of salt [2].

## Are heterocyclic amines involved in human carcinogenesis?

### Background

Many epidemiological studies have been published on the relationship between meat consumption and colorectal cancer development. In Japan, colorectal cancer has the highest mortality in women and the third highest in men in 2014. As shown in Table 1, the causes of colorectal cancer are attributed to alcohol drinking, tobacco smoking, overweight and insufficient physical activity. However, the causes of about 40 % of colorectal cancers in men and 85 % in women are not known.

HCAs are strong mutagens present in cooked meat/fish, and are carcinogenic in experimental animals, such as mice, rats and monkeys. Based on the experimental animal studies, it was suggested that carcinogenic activities and the target organs are different in different species. For instance, TD_50_ of 2-amino-3,4-dimethylimidazo[4,5-*f*]quinoline (MeIQ) was 0.1 mg/kg/day in rats and 8.4 mg/kg/day in mice: corresponding to an 84-fold difference between these species. The TD_50_ indicates the daily dose level that produces tumors in 50 % of tested rodents during their entire lifespan, which is considered to be 2 years for mice and rats. Furthermore, 2-amino-1-methyl-6-phenylimidazo [4,5-*b*]pyridine (PhIP) induced cancer in colon, breast, prostate, and the hematopoietic system in rats, but only in the hematopoietic system in mice [3].

Although many epidemiological studies on HCAs as causative agents in human carcinogenesis have been carried out, clear positive results were barely reported before 2000. However, many positive reports are now available for not only colorectal adenomatogenesis, but also for pancreas, bladder and renal cell carcinogenesis.

### Colorectal cancer

Budhathoki et al. [12] studied the involvement of HCAs in colorectal adenoma (established precursor lesion of cancer) risk in the Japanese population, by a case-control study. The subjects comprised 738 adenoma cases detected by colonoscopy and 697 cases of negative controls. They had no histories of colorectal adenomas, malignant neoplasms, ulcerative colitis, Crohn disease, familial adenomatous polyposis, carcinoid tumors or colectomy. Based on a food frequency questionnaire, one of the author’s (Tsugane’s) group estimated the contents of HCAs in meat, using their own database [13].

In men, BMI, alcohol intake, tobacco smoking and nonsteroidal anti-inflammatory drug (NSAID) use showed significant differences between cases and controls with *P*-values of less than 0.02. Red meat intake did not produce a significant difference between the cases and controls in either men or women. In women, the MeIQ intake was significantly different between cases and controls with a *P*-value of 0.005. The total HCA intake also showed a significant difference with a *P*-value of 0.02. Other positive factors in women were parenteral colorectal cancer and total meat intake, both with a *P*-value of 0.02. In women, the odds ratios in quartiles for MeIQ-intake showed a *P*-trend of 0.01 (Table 3). Supportive evidence is available for the increased risk of colorectal adenoma in women with MeIQ [14].

**Table 3:** Effect of HCAs in colon carcinogenesis in case control studies^a^

Author (ref)	Country/year	No. case/control	HCA	OR	P-trend
Budhathoki S et al. [12]	Japan, 2015	738/697	MeIQ	2.80 (qu)^b^	0.005^c^
			HCA	1.73	0.03^c^
Fu Z et al. [16]	USA, 2011	2,543/3764	PhIP	1.3 (qu)	0.005
			MeIQx	1.4	0.062
Helmus DS et al. [17]	USA, 2013	1062/1645	MeIQx	1.73 (qu)	<0.0001
			DiMeIQx	1.88	<0.0001
			red meat PhIP	1.38	0.009
			white meat PhIP	0.86	0.180
Barbir A et al. [18]	Switzerland, 2012	413/796	PhIP	1.75 (qi)	0.006
			MeIQx	1.57	0.022
			DiMeIQx	1.46	0.045
Gilsing AM et al. [19]	USA, 2012	1243/1419	MeQx	1.43 (t)	0.007
			MeIQx-NAT1		0.001^d^

^a^Detection of adenoma by colonoscopy, ^b^ *qu* quartiles, *qi* quintiles

^c^For women but not for men, ^d^Interaction with NAT1

In the Budhathoki’s study [12] fish was the largest contributor in both men and women to the intake of three HCAs, namely PhIP, MeIQ and 2-amino-3,8-dimethylimidazo[4,5-*f*]quinoxaline (MeIQx), however, no significant results were obtained in men with any of these HCAs. Further confirmation is needed.

MeIQ was not detected in any heated meat samples evaluated by Sinha and her colleagues [15], and therefore it is not included in the CHARRED database, which is frequently used by many epidemiologists to evaluate the role of HCAs in human carcinogenesis.

Four other studies, using the CHARRED data base, demonstrated risks of HCA compounds in human colon carcinogenesis, and the results are summarized in Table 3. All these were case/control studies and used colonoscopy for detecting adenomas. Three studies [16–18] detected an association of risk for adenomatogenesis with PhIP intake, and all four studies [16–19] detected an association with MeIQx intake, and two studies [17, 18] detected an association with 2-amino-3,4,8-trimethylimidazo[4,5-*f*]quinoxaline (DiMeIQx) intake.

Budhathoki et al. [12] studied the role of the NAT2 (*N*-acetyltransferase 2) acetylation genotype itself in colon carcinogenesis, and also in association with HCAs or MeIQ intake, and found no effects of the NAT2 genotype. Gilsing et al. [19] reported on an interaction of NAT1 (*N*-acetyltransferase 1) with MeIQx intake (Table 3).

### Pancreatic cancer

Involvement of HCAs in pancreatic cancer was demonstrated in four studies, one was a case control and three were cohort studies. All studies were performed in the United States, and the CHARRED database [15] was used to estimate the amounts of HCAs in charred meat. Anderson et al. analyzed HCA and benzo[*a*]pyrene (B[*a*]P) effects on pancreatic cancer development in the case control study, using 193 cases and 674 controls, diagnosed between 1994 and 1998 [20]. PhIP, DiMeIQx and B[*a*]P showed positive results with *P-*trend values in quintiles of 0.006, 0.029 and 0.050, respectively. They also demonstrated involvement of DiMeQx and MeIQx with pancreatic cancer in a cohort study. Among the 62,581 subjects, 248 cases of exocrine pancreatic cancer were confirmed. DiMeIQx and MeIQx, but not B[*a*]P or PhIP, showed *P*-trend values of 0.01–0.03, in their quintiles. Hazard ratios of the highest quintiles of DiMeIQx and MeiQx were around 1.8.

In a cohort study using 317,371 cohorts and 459 cases, Stolzenberg-Solomon et al. [21] showed associations of total, red and high-temperature-cooked meat intake with pancreatic cancer among men, with a hazard ratio (fifth versus first quintile) of 1.41 with a *P*-trend of 0.001, a hazard ratio of 1.42 with a *P*-trend of 0.01 and a hazard ratio of 1.52 with a *P*-trend of 0.005, respectively, but an association was not found in women. DiMeIQx showed a hazard ratio (fifth versus first quintile) of 1.29 with a *P*-trend value of 0.006 only in men and women combined. Associations were not demonstrated with MeIQx, PhIP or B[*a*]P. Li et al. [22] demonstrated association of DiMeIQx and B[*a*]P with pancreatic cancer in a case/control study of 626 cases and 530 controls.

### Other cancers

Lin et al. [23] demonstrated risks from HCAs in bladder carcinogenesis, using 884 cases of bladder cancer and 878 healthy controls, recruited from 1999 to 2009 in the United States. They demonstrated positive associations of bladder cancer with intakes of total HCA, PhIP, MeIQx, and DiMeIQx. Their *P*-trend values for each quartiles were 0.003, 0.004, <0.001 and <0.001, respectively.

Daniel et al. [24] analyzed roles of HCAs in renal cell carcinogenesis in a United States cohort study using 492,186 cohorts with 1,814 renal cell carcinomas cases. Among these, 115 were papillary cell carcinomas. They demonstrated that the hazard ratios of B[*a*]P, PhIP and MeIQx in renal papillary cell carcinomas were 2.17, 2.17 and 1.76 fold at the highest/lowest quintiles, respectively, with *P*-trends of 0.002, 0.03 and 0.03, respectively. However, no associations were observed for the clear cell subtype of renal cancer.

## Discussion

Chronological changes in cancer mortality indicate that in general, cancer death in young individuals has been decreasing, although the overall cancer mortality is increasing. Death at a young age from stomach cancers and esophagus cancer in men and women, and liver cancer in men has decreased dramatically between 1965 and 2013 [1], although the incidence of most types of cancer in old individuals is increasing [1]. By living an ideal life style and considering the information gained from the epidemiological studies [2], cancer deaths in young individuals will be further prevented. However, accumulation of DNA replication errors, and mutations induced by mutagens present in foods as minor components will accumulate during the course of the lifetime, so old-age cancer may not be preventable. The epidemiological study on causes of cancer in Japan, presented in this article [2], does not include age as a cause of cancer. If it is taken into consideration, important new information will be provided.

**Fig. 4:**
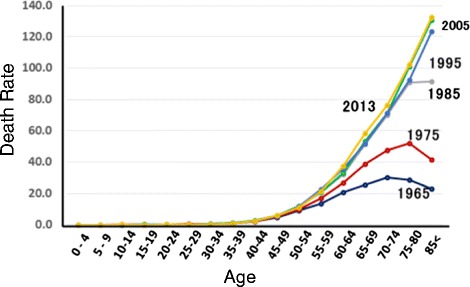
Trend in age-specific mortality rate of pancreatic cancer in Japan [1]

**Fig. 5:**
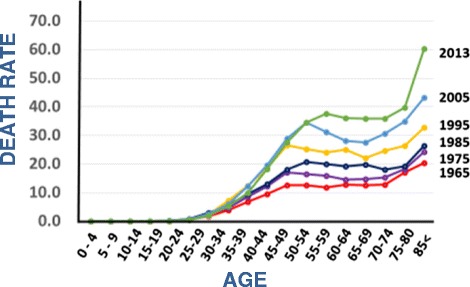
Trend in age-specific mortality rate of breast cancer in Japan [1]

As shown in Figs. 4 and 5 the mortalities of pancreatic cancer and breast cancer are increasing at all ages. Furthermore, attention should be paid to the high mortality in young individuals of both cancers. Tobacco smoking is involved in pancreatic cancer deaths with PAFs of 23.9 % in men and 9.5 % in women, and overweight and obesity (BMI ≥25) is associated with a PAF of 2.4 % in women. However, major causes of these cancers have not yet been clarified.

HCAs have been shown to be involved in various cancers in recent epidemiological studies. Establishment of the CHARRED data base [15] might make it possible to analyze the effects of HCAs in humans. Meanwhile, one of the authors’ (Tsugane’s) group is using a different database [13] that includes MeIQ. This compound is not included in the CHARRED data base. Since HCAs were found using the mutagenicity in *S. typhimurium* as a marker, and almost all people intake them almost every days although their amounts are not so high, a clarification of roles of HCAs in human carcinogenesis is anticipated.

## Conclusion

Cancer mortality rate is increasing year by year in Japan. However, mortality rate among young people is decreasing in men and women as a whole, although some kind of cancer, such as pancreatic cancer and breast cancer are increasing. Analysis on attributable causes indicated 50 % of cancer can further be preventable. Prevention from infection and refraining from tobacco smoking will reduce cancer mortality by about 40 %. Food borne mutagenic/carcinogenic heterocyclic amines were indicated to be involved in colorectal, pancreas, bladder and renal cancer, by epidemiological studies. Clarification of rolls of age factor and mutagens in foods are anticipated to be clarified.
